# The antimicrobial and cytotoxic effects of a copper-loaded zinc oxide phosphate cement

**DOI:** 10.1007/s00784-020-03257-w

**Published:** 2020-03-20

**Authors:** Torsten Wassmann, Andrea Schubert, Felix Malinski, Martin Rosentritt, Sebastian Krohn, Kirsten Techmer, Ralf Bürgers

**Affiliations:** 1 Department of Prosthodontics, University Medical Center Goettingen, Goettingen, Germany; 2 Department of Prosthetic Dentistry, University Medical Center, Regensburg, Germany; 3 Department of Crystallography, Georg-August-University Goettingen, Goettingen, Germany

**Keywords:** Dental luting material, Zinc oxide phosphate cement, Copper, *Candida albicans*, *Streptococcus sanguinis*, Biofilm, Cytotoxicity

## Abstract

**Objectives:**

Evidence about modifications of dental luting materials to minimize biological failure at the “marginal gap” between teeth and fixed prosthodontics is scarce. We compared a copper-modified (Co-ZOP) and a conventional zinc oxide phosphate cement (ZOP) in terms of antimicrobial and cytotoxic potentials in vitro and in vivo.

**Materials and methods:**

Specimens of ZOP and Co-ZOP were characterized by the mean arithmetic roughness (Ra) and surface free energy (SFE). Powder components were examined using scanning electron microscopy (SEM). Energy-dispersive X-ray spectroscopy (EDX) showed elemental material compositions. In vitro microbial adhesion was shown using SEM, luminescence, and fluorescence assays. CCK-8 assays of mouse fibroblasts (L929) and human gingival fibroblasts (GF-1) were performed after 6, 24, and 48 h of specimen incubation. In vivo, ZOP and Co-ZOP specimens were applied intraorally for 12 h; biofilm accumulation was shown using SEM.

**Results:**

Ra of ZOP and Co-ZOP showed no significant differences; SFE was significantly higher for Co-ZOP. EDX exhibited minor copper radiation for Co-ZOP, none for ZOP. In vitro fungal adhesion to Co-ZOP was significantly higher than to ZOP; in vitro streptococcal adhesion, cytotoxicity, and in vivo biofilm formation were not significantly different.

**Conclusions:**

Co-ZOP showed low surface allocations of copper with no improved antimicrobial properties compared with conventional ZOP in vitro or in vivo.

**Clinical relevance:**

Antimicrobial effects and low cytotoxicity of biomaterials are important for the clinical outcome. Based on our in vitro and in vivo results, no clinical recommendation can be given for the tested Co-ZOP.

## Introduction

Dental luting materials are used to attach fixed prosthodontics such as crowns and bridges definitively or provisionally on natural teeth and implants [1]. To leave space for the luting material, crowns and bridges are designed with a defined distance to the teeth, the so-called cement gap or internal fit. Although essential, the cement gap at the interface between the tooth and the margin of the crown (“marginal gap”) is a major trigger for biological failure because it entails a structured predilection area for colonization by a multitude of microorganisms whose initial and reversible attachment eventually leads to a complex biofilm [2, 3]. Depending on their specific microbial composition and other modulating factors, biofilms maintain, enhance, and finally cause diseases such as periodontitis, peri-implantitis, and secondary caries at the tooth-biomaterial interface [4]. The latter phenomenon is known as one of the main reasons for the failure of fixed dental restorations—enhancing the relevance of biofilm formation at the cement gap [5]. To prevent secondary caries, several efforts have been undertaken, including minimization of the cement gap and improvement of oral hygiene. Moreover, the luting material itself has been modified to become less susceptible to biofilm formation or even to gain antimicrobial properties [6–9]. For example, nanoglasses, chlorhexidine, chitosan, and metallic nanoparticles have been applied as antibacterial and antifungal additives [10]. Corresponding in vitro or in vivo trials showed differing effectiveness of these modifications, but none of these antimicrobial alterations has prevailed over the others or is actually widely used in dentistry [11–15]. The luting materials tested in the present study are zinc oxide phosphate cements (ZOP), representing the most common conventional luting material in dentistry [1]. While studies on most modified luting materials are in early test phases, both the conventional ZOP (Hoffmann’s cement, Hoffmann’s Dental Manufaktur, Berlin, Germany) and the copper-modified ZOP (Co-ZOP, Hoffmann’s copper cement, Hoffmann’s Dental Manufaktur) in the present study are commercially available and in clinical use. The tested copper additive is proclaimed to have antimicrobial potential, which in turn might reduce secondary caries and gingival inflammation. However, to our knowledge, there are no in vitro or in vivo studies investigating the promoted potential. Copper has been used as an antimicrobial agent in medicine for centuries; copper ions are capable of killing bacteria on direct contact [16, 17]. Similar effects were observed for antifungal and even antiviral use [18].

This study tested the hypothesis that the antimicrobial and antifungal activities as well as the cytotoxic potential of a Co-ZOP (Hoffmann’s copper cement) in vitro and in vivo are higher than those of a conventional ZOP (Hoffmann’s cement). The aim of the present study was to examine whether a clinical recommendation for the use of the tested copper-loaded ZOP can be given.

## Materials and methods

### Preparation and characterization of test materials

#### Specimen preparation

**Fig. 1:**
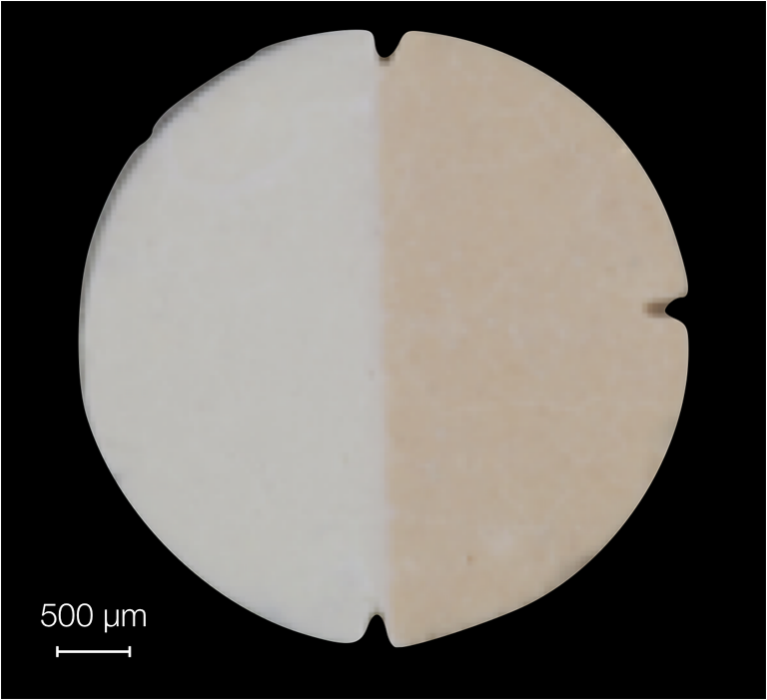
Enlarged depiction of a split specimen before in vivo testing. The left side shows the conventional zinc oxide phosphate cement, and the right side shows the copper-loaded zinc oxide phosphate cement. Three tags were used to facilitate orientation during quantification of biofilm-covered areas

The conventional ZOP (Hoffmann’s cement) and the Co-ZOP (Hoffmann’s copper cement) were both commercially available. For the in vitro testing, solid cylindrical specimens of both cements (diameter 10 mm, height 1 mm) were prepared according to the manufacturer’s instructions using custom-built silicone molds. After 24 h, the specimens were polished with grinding paper (grain 1200, Leco Corporation, St. Joseph, MI, USA). For in vivo biofilm accumulation, ZOP, Co-ZOP, and split cylindrical specimens (diameter 4 mm, height 1 mm) were similarly prepared. Specimens were smoothed from both sides using the same grinding paper. On split specimens, the dividing line between both cements and the external margin of the conventional ZOP was marked with three tags to facilitate differentiation of both cements in SEM (Fig. 1). All specimens were stored in *aqua dest*. for 3 days and sterilized by UV radiation prior to the in vivo testing.

#### Surface characteristics

The mean arithmetic roughness (Ra) was measured and calculated automatically with a perthometer (Perthometer S6P6, Feinprüf Perthen, Goettingen, Germany) using the stylus method [19]. Three separate measurements were performed with eight specimens of each group. The surface free energy (SFE) and its polar and disperse components were determined by contact angle measurement using the sessile drop method (Goniometer G1, ERNA, Tokyo, Japan) [19]. The analysis of the results was carried out via software (OCA 15 plus, Dataphysics Instruments, Filderstadt, Germany). For each test group, four specimens were tested at three different sites each.

#### EDX

Elemental analysis of the powder components and the cured specimen surfaces of both test cements was performed using EDX (Quanta FEG 200, FEI Company, Hillsboro, OR, USA). Spectrum analysis was used to display the elemental composition of the materials; mapping visualized the two-dimensional allocation of zinc and copper.

#### SEM

For the in vitro trials, SEM (Cambridge S240, Cambridge Instruments, Nussloch, Germany) with magnification factors from × 24 up to × 4000 was used at a tube voltage of 10 kV to show microbial adhesion. For the in vivo trials, different SEM (Quanta FEG 200, FEI Company) with magnifications up to × 12,000 were used at tube voltages from 1 to 3 kV to show biofilm accumulation.

### In vitro microbial adhesion

#### Microbial cultures

Cultures of *Streptococcus sanguinis* (Leibniz Institute DSMZ - Deutsche Sammlung von Mikroorganismen und Zellkulturen GmbH, DSMZ No. 20068) and *Candida albicans* (DSMZ No. 1386) were prepared from cryocultures. Week cultures were prepared by inoculating the thawed culture into tryptase soy yeast extract medium (DSMZ medium No. 92) for *S. sanguinis* and universal medium for yeast (DSMZ medium No. 186) for *C. albicans* and subsequent cultivation for 24 h at a temperature of 37 °C in an incubator. Twenty-four h before the preparation of the test cultures, the procedure was repeated with 10 μl of the suspension to obtain a vital and growing culture. For the experiments, the pH values were determined. The optical density was adjusted to a value of 0.3 A.

#### Luminescence assay

The initial adhesion of *C. albicans* was measured using a bioluminescence-based assay (ViaLight Buffer, LT27-079 Assay buffer, ViaLightPlusCell Proliferation and Cytotoxicity assay, Lonza, Basel, Switzerland) as described before [20]. In brief, specimens were mounted in 48-well plates using dental silicone (previous testing showed no negative side effects on microbial adhesion, data not shown). For each run and subgroup, 12 specimens were required; two test specimens were used as controls. A safety cabinet’s UV spotlight was used for disinfection of the specimens by irradiation for 1.5 h. Luminescence measurement was carried out with a plate reader (Fluostar Optima, BMG LabTech, Offenburg, Germany) at a preset gain of 4000.

#### Fluorescence assay

The initial adhesion of *S. sanguinis* was measured by a resazurin assay (resazurin salt, Sigma Aldrich, St. Louis, MO, USA) as previously described [21]. The test specimens were fixed in 48-well plates using silicone and disinfected by UV radiation. The adherence of the bacteria was measured on ten specimens, three test specimens were used as a color control, and one was used as a bacterial or negative control. To eliminate potential influences of autofluorescence, it was recorded and saved for a subsequent calculation.

### In vivo biofilm formation

The in vivo study plan was approved by the Ethics Committee of the Faculty of Medicine, University Medical Center Goettingen (application number 12-9-13). Ten women and ten men (age 26 ± 3.4 years) volunteered to participate in the study; all participants gave their written informed consent. The exclusion criteria were age under 18 years, antibiotic therapy in the last two months, xerostomia, or radiation therapy to the head or neck. Oral examination was carried out by an experienced dentist. All volunteers had excellent oral hygiene, no caries, and no periodontal infections (plaque indices < 10% and sulcus bleeding indices < 5%). Before biofilm testing, an overview image of each specimen was captured to document the output state (biofilm-free) using SEM (Quanta FEG 200, FEI Company, USA; magnification 50-fold to 60-fold).

All specimens were sterilized by UV radiation and fixed to individual removable acrylic upper jaw splints, as described before [22]. Specimens were positioned in the buccal region of the premolars and molars. Each volunteer had four specimens inserted, whereby group 1 had two ZOP specimens and two Co-ZOP specimens and group 2 had four split specimens. Splints were removed only for oral hygiene and food or beverage consumption. After 12 h, the plaque-covered specimens were removed from the splints and immediately processed for SEM imaging. All specimens were transferred to well plates and washed in PBS to remove non-adherent cells. SEM images were captured according to the procedure described above. Plaque-covered areas were significantly darker and richer than uncovered areas. Images were converted into 8-bit images and transformed into false-color images by a standardized threshold. The biofilm coverage (as a percentage of total surface) was calculated quantitatively using surface analysis software (ImageJ 1.48, NIH, MD, USA), and the orientation tags were excluded from the calculations. To confirm the biofilm adhesion, micrographs with higher magnification (up to 12,000-fold) were taken.

### In vitro cytotoxicity

#### Cell cultures

Mouse fibroblasts (L929) comply with the ISO 10993-5 standards for cytotoxicity testing and were obtained commercially (L929, Nr. 85011425, Cell Line from mouse, Sigma Aldrich, Munich, Germany). Immortalized human gingival fibroblasts (GF1) were established by the group for oral biology and tissue regeneration at our department (ethic vote No. 16/6/2009). Cell isolation, immortalization, and cultivation were performed as previously described [23].

#### Colorimetric assay

L929 cells were cultured in DMEM (Dulbecco’s Modified Eagle’s Medium, Thermo Fisher Scientific, Waltham, MA, USA) with 10% FCS (FCS, Invitrogen, Darmstadt, Germany) and 1% penicillin-streptomycin (Fisher Scientific, Schwerte, Germany) at 37 °C, 5% CO_2,_ and 95% relative humidity. The GF1 cells were cultured in DMEM + GlutaMax (Dulbecco’s Modified Eagle’s Medium + GlutaMax, Thermo Fisher Scientific, Waltham, MA, USA), 10% FCS (FCS, Invitrogen, Darmstadt, Germany), and 50 μg/ml gentamycin (PromoCell, Heidelberg, Germany) at 37 °C with 5% CO_2_ and 95% relative humidity.

The kit used to determine cytotoxicity (Cell Counting Kit 8, CCK-8, Dojindo Molecular Technologies, Kumamamoto, Japan) is based on a highly water soluble, nontoxic tetrazolium chloride (2-(2-methoxy-4-nitrophenyl)-3-(4-nitrophenyl)-5-(2,4-disulfophenyl)-2H-tetrazolium, monosodium salt, WST-8). Its nontoxic properties allow for multiple measurements at different times without cell damage.

The specimens were fixed to the well bottoms of 48-well plates using silicone; disinfection was carried out by UV radiation for 1.5 h. A total of nine sample bodies were used per test run and sample group, followed by four wells with a silicone layer and cells as positive controls. A total of 10,000 cells in 0.5 ml of the culture medium were seeded into each well. Successful adherence was determined via light microscopy. Afterwards, the medium was removed from the wells and replaced by 0.5 ml of the CCK-8 detection solution at a dilution of 1:10 in culture medium. After 6 h of incubation, 100 μl of the supernatant was transferred to a 96-well plate. Wells were washed twice with PBS to remove the remaining sample solution before 0.5 ml of the cell culture medium was added. After 24 h and 48 h of incubation, measurements were repeated. Colorimetry was performed using a plate reader (Fluostar Optima, BMG Labtech, Offenburg, Germany, in absorption measurement mode) at 450 nm and a reference wavelength of 650 nm.

### Statistics

Statistical analyses were performed using the R (R, version 3.0.2, www.r-project.org) and SPSS (IBM SPSS Statistics for Mac, 24th version for 64-bit-systems, IBM, Armonk, NY, USA). The overall level for significance was set to *α* = 0.05.

First, the results of the in vitro trials were tested for variance homogeneity (Levene test) and normal distribution (Kolmogorov-Smirnov test). If both were given, a one-way ANOVA followed, and in case of significant differences, Tukey-HSD post hoc testing was performed. If there was no normal distribution or variance homogeneity, Games-Howell post hoc tests were carried out.

The in vivo results were analyzed in two different ways: the parameters “age” and “plaque” (percent of covered surface area) were described using means and standard deviations. For the parameter “plaque,” the influences of the type of cement, age, sex, and their interactions with the specimens were examined by means of a general linear model for repeated measurements. The order of the parameters in the multivariable model depended on the results of the univariate analysis. The parameters with the lowest *p* values were first supplied to the multivariate model.

## Results

### Physicochemical characterization of the test materials

#### SEM of surfaces

**Fig. 2:**
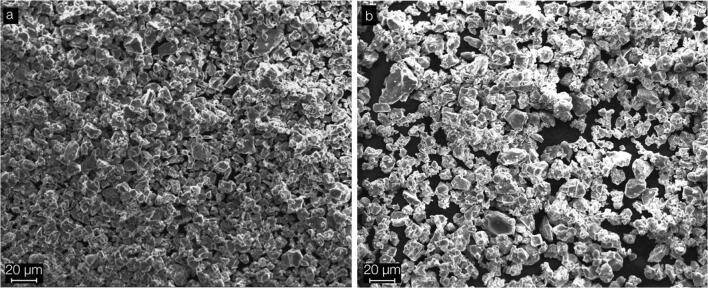
SEM images of the powder components of (**a**) ZOP and (**b**) Co-ZOP. The grain sizes of both powders are comparable. The black areas between the particles represent carbonic carrier foils of the sample holder

Using SEM imaging, the powder components of both tested materials showed a comparable finely grained, homogenous appearance (Fig. 2).

#### Surface characteristics

There were no significant differences in Ra values for both tested cements (*p* = 1.0). The measured mean values were Ra = 1.0 μm for ZOP and Ra = 1.02 μm for Co-ZOP. The SFE measurements showed statistically significant differences between the test groups (*p* < 0.001), i.e., SFE values of 57.2 mN/m for ZOP and 74.3 mN/m for Co-ZOP.

#### EDX

**Fig. 3:**
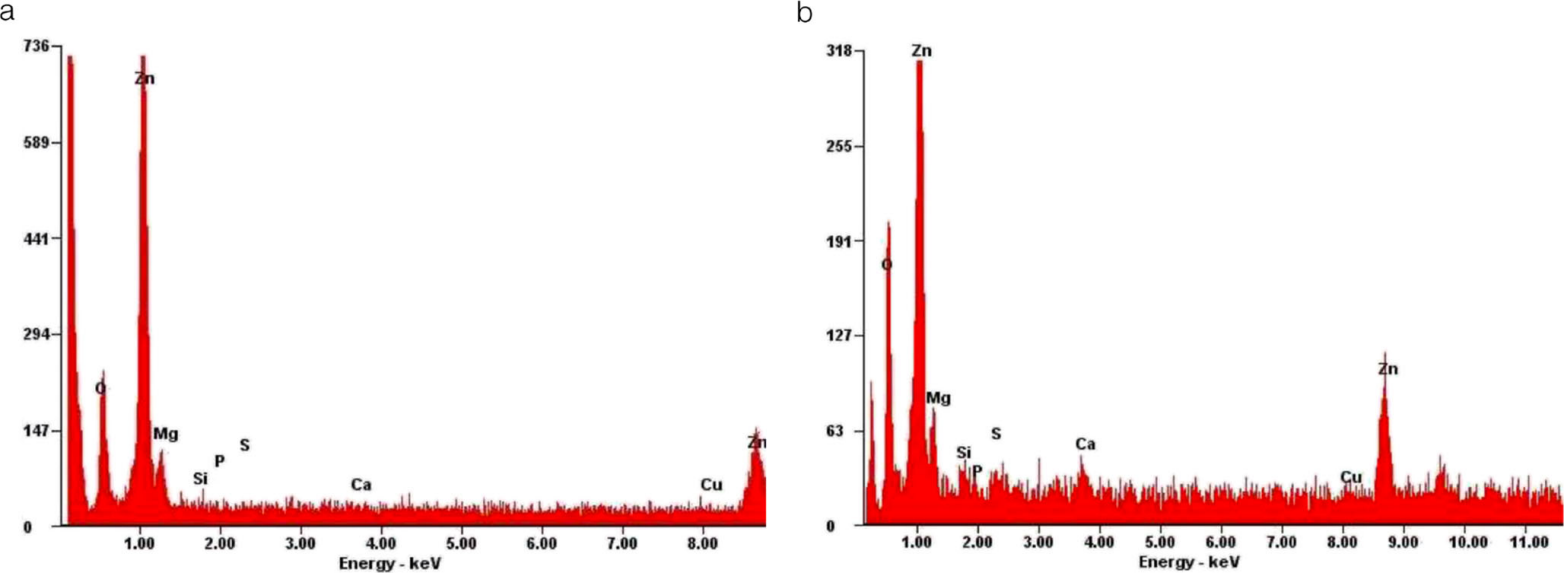
EDX spectra with signal intensities of the powder components of (**a**) ZOP and (**b**) Co-ZOP. Both zinc oxide phosphate cements show significant peaks for zinc (Zn), oxygen (O), and carbon (C), but not for copper. The high carbon peak is caused by the carbonaceous base film of the sample holder beneath the test powders

**Fig. 4:**
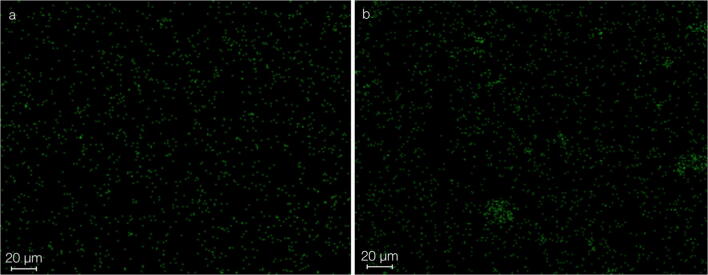
EDX mapping for copper in both zinc oxide phosphate cements. **a** ZOP: no accumulation of copper is detectable; radiation is unspecific and heterogeneously distributed. **b** Co-ZOP: small areas with enhanced copper radiation are detectable; these areas are associated with cement particles

**Fig. 5:**
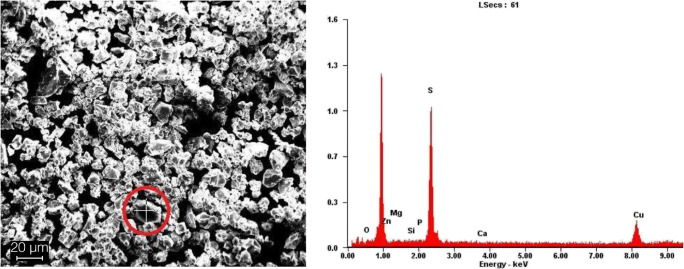
SEM image of the Co-ZOP (left side) with a copper-bearing particle (red circle) that was used for EDX point analysis of the local components (right side). Considerable peaks for zinc (Zn), sulfur (S), and copper (Cu) were detected

The spectra of the EDX analysis exhibited significant peaks for zinc, oxygen, and carbon for both tested materials (Fig. 3). The high carbon peak was caused by the carbonaceous base film of the sample holder beneath the test powders (Fig. 2). In both test materials, no significant peak for copper was found. EDX mapping showed no enhanced accumulation of copper for the ZOP specimens, and only unspecific and heterogeneously distributed radiation was detected. The mapping for Co-ZOP exhibited occasional small areas with enhanced copper radiation; these areas were clearly associated with specific cement particles (Fig. 4). For a detailed analysis of these copper-bearing particles, EDX point analysis was carried out and compared with adjacent areas. Considerable peaks for zinc, sulfur, and copper were detected (Fig. 5). No copper peaks were found on the ZOP particles via EDX point analysis.

### In vitro microbial adhesion

#### SEM imaging

**Fig. 6:**
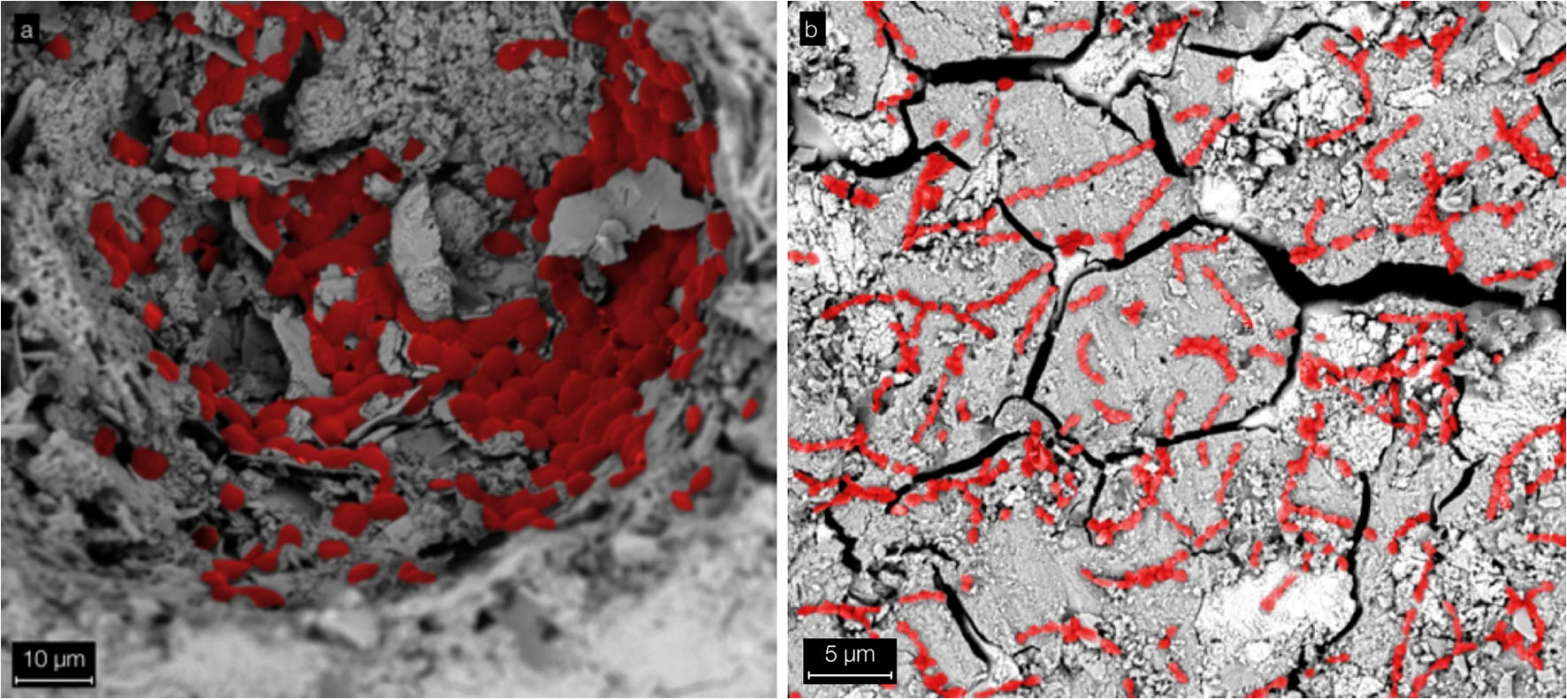
SEM images (manually colored) of microbial adhesion. **a** *C. albicans* cells accumulate in an indentation of a ZOP specimen. **b** *S. sanguinis* cells form chains on a Co-ZOP specimen

Monolayer biofilms with statistically distributed streptococci and fungi were found on both tested surfaces after in vitro adhesion (Fig. 6).

#### Adhesion assays

The in vitro adhesion of *C. albicans* was significantly higher (*p* < 0.0001) for Co-ZOP (60 ± 21.9 [relative luminescence unit = rlu]) than for ZOP (33.15 ± 12.2 [rlu]). The adhesion of *S. sanguinis* showed no significant differences (*p* = 0.865) between ZOP (37,383.2 ± 15,659.5 [relative fluorescence unit = rfu]) and Co-ZOP (33,812.9 ± 16,190.9 [rfu]).

### In vivo biofilm formation

**Fig. 7:**
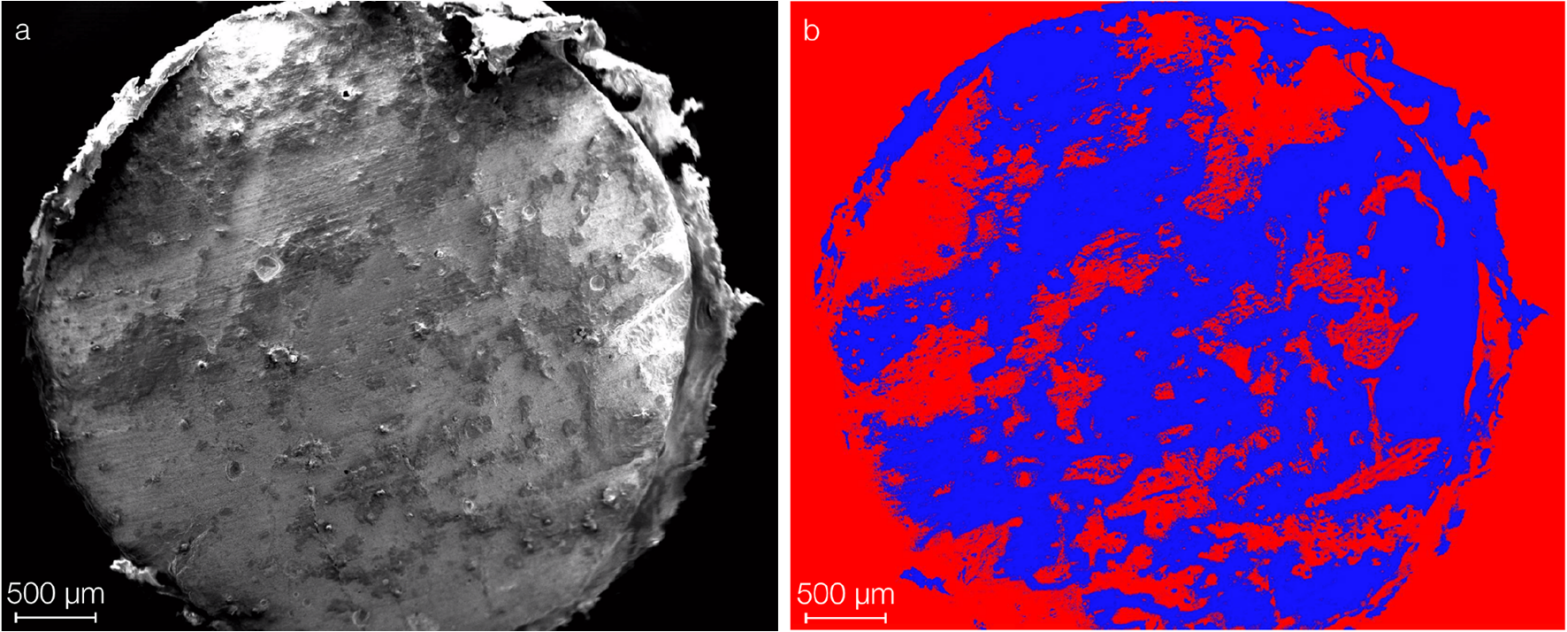
Enlarged depiction of a ZOP specimen after in vivo testing. **a** SEM imaging shows biofilm coverage. **b** The false-color image of the same specimen was used for biofilm quantification. Biofilm coverage on the ZOP and the Co-ZOP were not significantly different (*p* = 0.4102). There was no correlation between biofilm accumulation and sex (*p* = 0.689) or age (*p* = 0.1406)

Figure 7 shows a representative SEM image of the biofilm accumulation on a ZOP specimen and the corresponding false-color image that was used to quantify the biofilm. The results for biofilm accumulation in the in vivo study were as follows: ZOP, 47.3% ± 23.5 (males 51.8% ± 27.3, females 42.8% ± 18.6); Co-ZOP, 50.9% ± 22.1 (males 53.9% ± 22.1, females 47.9% ± 23.2). There was no statistically significant difference between biofilm accumulation on specimens incorporated in male or female participants (*p* = 0.689). We did not find any correlation between participants’ age and biofilm accumulation (*p* = 0.1406). Differences between the biofilm coverage on the ZOP and the Co-ZOP were not significant (*p* = 0.4102).

### In vitro cytotoxicity

**Fig. 8:**
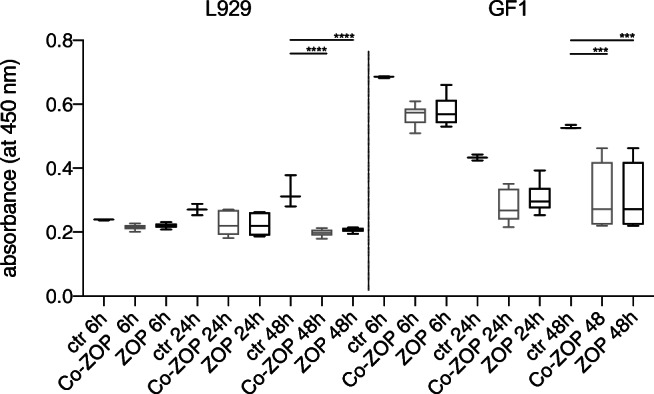
Results of the CCK-8 assays to test cytotoxicity of both zinc oxide phosphate cements (*n* = 48) to L929 and GF1 cells. After 6 h, 24 h, and 48 h, one-way ANOVA showed no significant differences in cytotoxicity to L929 and GF1 cells for ZOP and Co-ZOP (*p* > 0.9). After 48 h, a significantly higher cytotoxic effect was shown for both zinc oxide phosphate cements compared with the control group (**** indicates *p* < 0.0001, *** indicates *p* < 0.001). ctr, control group

After 6 h, 24 h, and 48 h, ZOP and Co-ZOP showed no significant differences in cytotoxicity to L929 and GF1 cells (*p* > 0.9). After 48 h, a significantly higher cytotoxic effect was shown for both test groups compared with the control group (*p* < 0.05) (Fig. 8).

## Discussion

In the present study, we tested the hypothesis that the antimicrobial and antifungal activities as well as the cytotoxic potential of a Co-ZOP in vitro and in vivo are higher than those of a conventional ZOP. *S. sanguinis*, a gram-positive, facultatively anaerobic bacterium representing an early colonizer during biofilm development, and *C. albicans*, a potentially human pathogenic yeast fungus, were used to test antimicrobial and antifungal effects. Cytotoxic effects were tested using L929 and GF1 cells. Furthermore, physicochemical parameters were investigated to determine possible influences of surface roughness and SFE. Additionally, the copper surface concentration and allocation were determined and correlated to possible biological effects. Finally, in vivo biofilm formation was examined among a population of healthy probands wearing occlusal splints with test specimens. Due to the results of the present investigations, our initial hypothesis must be denied.

While in vitro experiments on initial microbial adhesion can minimize confounders by standardization, in vivo settings allow the inclusion of host factors, such as individual composition of saliva, the varying oral microbiome, and the immune system [24–26]. Regarding cytotoxicity tests, a stepwise approach from simple cell culture models to in vivo models is the common procedure for testing new biomaterials [27]. Although not all tests on potentially harmful substances and biomaterials are transferable to humans, potential health risks can still be identified with great predictability [28–30]. Hence, a combination of in vitro and in vivo testing was performed in this study, based on studies with comparable questions and settings [10, 31, 32]. Surprisingly, no in vivo or in vitro studies on antimicrobial effects (and possible cytotoxic side effects) have been published for the new copper-loaded luting material, even though copper as an additive is known for both antibacterial and cytotoxic properties [16, 17, 20]. Since both ZOPs are approved for clinical use and fulfill the legal provisions, in vivo trials had minimized risks for the volunteers. We intended to deliver data on the biological effects of this novel “anti-microbial” cement in addition and to evaluate the clinical appropriateness of mixing copper into conventional ZOP.

The measurement of the physicochemical parameters showed no significant difference for the surface topographies, represented by Ra values. For titanium surfaces, an Ra threshold value of 0.2 μm may be assumed, below which microbial adhesion does not further decrease [33]. The Ra values in the present study were clearly above the threshold value: as the material properties and the specimen manufacturing resulted in porosities, surface polishing was limited. However, for the interpretation of the results, the Ra values were negligible due to the lack of significant differences.

The influence of SFE on microbial adhesion has been demonstrated in various studies and on different surfaces [33–35]. Depending on the material and the microbial species, the SFE or the roughness of a specific substratum may influence the microbial adhesion more significantly [36]. In the oral cavity, a stronger influence of the surface topography and the roughness is assumed [37–39]. Even if a statistically significant difference in SFE values was found, both materials exhibited similar low surface free energies from a clinical perspective [40]. Consequently, the statistically significant differences of the SFE of the two test materials can be neglected; the absence of measurable significant differences in the biological interactions of the ZOP reinforced this.

In the literature, “contact killing” abilities of copper are often described, but the underlying mechanisms are not yet fully understood [17, 41, 42]. The primary effect seems to be caused by damage to the cell membranes, while DNA damage occurs later [43–45]. This finding applies to both dry and moist copper surfaces [42, 46]. The efficacy of copper depends on its appearance, whereby certain copper ions (Cu_2_O) appear to be superior to others (Cu0) [41]. In addition, the surface structure and distribution of nanoparticulate copper appear to have a significant influence on the antimicrobial effects of copper surfaces [47]. Contrary to these findings, a significant increase in the initial adhesion of *C. albicans* to Co-ZOP was observed. This result might be attributed to a tolerance of the yeast towards an environment with high copper values [11]. In fact, unlike bacteria, for which copper primarily acts as a stress factor, fungi show an enzymatic dependence on copper [48, 49]. *C. albicans* has even developed mechanisms to compensate for copper deficiency and to react positively to copper released during an immune response [50–53]. Thus, the interactions between *C. albicans* and copper in infections are complex and the subject of ongoing research [54]. A possible interpretation of the measured adhesion is the compensation of a relative copper deficiency by the low copper admixture of the Co-ZOP. The probability of undesirable cytotoxic side effects against human and animal cells increases with increasing copper concentrations in biomaterials [18, 55, 56]. Applied to the results presented here, the low surface allocation and localization of copper appears to be below the required effective amount as no antimicrobial or antifungal effect was observed, while a fungus-promoting effect was shown. However, the occurrence of negative side effects of copper seems unlikely for the same reasons. In summary, the addition of copper in low concentrations as given does not have any significant (in vitro and in vivo) effects on microorganisms (or cells) and must therefore be categorized as counterproductive, according to our results and after considering the limitations of our experimental design.

In further consideration, the observed cytotoxic effects for the characterization of the cements are more significant than the missing antimicrobial effects. Similar results have already been reported for other ZOPs [57–59]. The cytotoxic effects observed for various types of cements tend to decrease over time, with ZOP being an exception showing significant cytotoxic effects over time [57, 60, 61]. Possible causes for these effects, in addition to the initial acidic reaction, are the release of zinc or zinc oxides [62, 63]. The effects of zinc oxide nanoparticles are the subject of scientific research outside dental research [64, 65].

The results of the present study showed no antimicrobial effects of the copper additive. In contrast, an enhancing effect of copper on the initial adhesion of *C. albicans* was shown. Also, the cytotoxic potentials of both tested ZOP were comparable. Thus, we reject our initial hypothesis. Within the limitations of this study, clinically, it is not beneficial to use Co-ZOP instead of ZOP. The frequent use of ZOPs by dental practitioners for economic reasons should be reconsidered. Instead, alternatives like adhesives and glass ionomer cements that unite lower cytotoxic effects and satisfying clinical performance should be used. The results emphasize the need for a rigorous and independent review of biomaterials for their efficacy prior to approval and clinical use.

## Conclusion

Within the limitations of this study, there is no evidence for improved antibacterial or antifungal properties of the tested copper-loaded cement, either in vitro or in vivo. In fact, *C. albicans* showed a significantly higher initial adhesion to Co-ZOP compared with conventional ZOP. These findings may result from a low surface allocation of copper in the Co-ZOP. Furthermore, both tested cements exhibited significant cytotoxicity against human and animal cell cultures. Based on the results of this study, no clinical recommendation can be given for the use of the tested Co-ZOP.
